# Editorial: Rethinking the Failure to Replicate

**DOI:** 10.1523/ENEURO.0042-18.2018

**Published:** 2018-01-29

**Authors:** Christophe Bernard

When launched, *eNeuro* included different types of scientific papers, including the Failure to Replicate category. As scientists, we know that many papers cannot be replicated, for many different reasons, e.g., lack of statistical power, misinterpretation, experimental caveat, and even fraud. When such papers are published in high-profile journals, they can send research in the wrong direction and lead the field astray. When they become dogma, it becomes very difficult to propose alternate views, and the whole community suffers.

Many of us have experienced not being able to replicate the major results of a very influential study. But it is very difficult to publish such contradictory results. Since *eNeuro* exists to serve the scientific community, it made sense to include a Failure to Replicate category, and we have published several of such papers.

However, after a discussion with some in our community, we can see the unintended perception that Failure to Replicate reads like an accusation, and some readers may be tempted to jump to the conclusion that the original story was fraudulent.

There are many reasons why results cannot be replicated, even when using the exact same experimental conditions. This is part of scientific variability (a genetic drift, a modified antibody, etc.). It is inherent to the experimental method, as we cannot control all parameters.

Because we wish to keep a positive approach to science, the Failure to Replicate category will be merged into the New Research category. Of course, providing a venue to publish such papers remains an important part of *eNeuro*’s mission. These studies are part of the way science moves forward; they are scientific papers, which happen to question previously published results. But in general, the title of the paper says it all and is enough by itself, without the need to categorize it as a failure to replicate study.

Please share your thoughts with us at eNeuro@sfn.org.

Striving to move science forward.

